# *Blumea balsamifera*—A Phytochemical and Pharmacological Review

**DOI:** 10.3390/molecules19079453

**Published:** 2014-07-03

**Authors:** Yuxin Pang, Dan Wang, Zuowang Fan, Xiaolu Chen, Fulai Yu, Xuan Hu, Kai Wang, Lei Yuan

**Affiliations:** 1 Tropical Crops Genetic Resources Institute, Chinese Academy of Tropical Agricultural Sciences, Danzhou, Hainan 571737, China; E-Mails: wang_dan1414@163.com (D.W.); hillowchan@hotmail.com (X.C.); fulai.yu@163.com (F.Y.); 2 Key Laboratory of Crop Gene Resources and Germplasm Enhancement in Southern China, Danzhou, Hainan 571737, China; E-Mails: fujianfanzuowang@126.com (Z.F.); mchuxuan@163.com (X.H.); 3 Hainan Provincial Engineering Research Center for *Blumea balsamifera*, Danzhou, Hainan 571737, China; E-Mails: jimojijie29@163.com (K.W.); ylei1216@126.com (L.Y.); 4 School of Traditional Chinese Medicine, Guangdong Pharmaceutical University, Guangzhou, Guangdong 510006, China

**Keywords:** Traditional Chinese Medicines, *Blumea balsamifera*, sambong, herbal authentication, phytochemistry, biological activities

## Abstract

The main components of sambong (*Blumea balsamifera*) are listed in this article. The whole plant and its crude extracts, as well as its isolated constituents, display numerous biological activities, such as antitumor, hepatoprotective, superoxide radical scavenging, antioxidant, antimicrobial and anti-inflammation, anti-plasmodial, anti-tyrosinase, platelet aggregation, enhancing percutaneous penetration, wound healing, anti-obesity, along with disease and insect resistant activities. Although many experimental and biological studies have been carried out, some traditional uses such as rheumatism healing still need to be verified by scientific pharmacological studies, and further studies including phytochemical standardization and bioactivity authentication would be beneficial.

## 1. Introduction

Nowadays, herbal medicines are widely consumed and their sales have been rising significantly all over the world. According to the reports of the World Health Organization (WHO), to treat diseases over 80% of the populations in developing countries mainly rely on herbs, which are considered to be safer and more effective than synthetic drugs [1,2,3].

*Blumea balsamifera* (L.) DC. (Asteraceae), also known as sambong, has been used as medicine for thousands of years in Southeast Asia countries, such as China, Malaysia, Thailand, Vietnam, and Philippines. Sambong is the most important member of the genus *Blumea* and is an indigenous herb of tropical and subtropical Asia, especially in China. This plant grows on forest edges, under forests, river beds, valleys and grasses [4,5]. In China, it is generally a common used herb in the areas south of the Yangtze River, such as Hainan, Guizhou, Yunnan, and Guangdong provinces and Taiwan [6,7,8]. *B. balsamifera* is commonly called “Ainaxiang” and “Dafeng’ai” in Chinese and used as incense because it has a high level of essential oils [9]. It was originally recorded in “Bei Ji Qian Jin Yao Fang” in 652 by Sun Simiao. The whole plant or its leaves were used as a crude Chinese traditional medicinal material to treat eczema, dermatitis, beriberi, lumbago, menorrhagia, rheumatism, skin injury, and as an insecticide [10]. Bing Pian and Aipian are two important traditional Chinese medicines (TCMs) extracted from plants and have been used as one in prescriptions for centuries in China. Both of them mainly contain borneol and are similar in efficacy [11]. They are synonymous in the Chinese pharmaceutical industry nowadays. Before 2010, sambong was one of the most important plant sources for Bing Pian, but since 2010, the Pharmacopoeia of the People’s Republic of China records *B. balsamifera* as the only plant source for Aipian [11], with a consistent efficacy with *B. balsamifera* medicinal materials, which could induce resuscitation, clear heat, and relieve pain. Recently, extracts of its leaves have been verified do display various new physiological activities, such as antitumor [12,13,14], antifungal [13,15], radical-scavenging [16], and anti-obesity properties [17]. The main active compound is L-borneol, which was characterized by a high volatility. Besides, essential oils, flavonoids, and terpenoids with several different biological activities were also reported [18]. These studies could explain why this plant has multiple pharmacological effects.

In this review, botanical descriptions, herbal authentications, and phytochemical constituents of *B. balsamifera* are covered. In addition, the previous *in vitro* and *in vivo* studies conducted on its biological activities are reviewed, concentrating on antitumor, hepatoprotective, superoxide radical scavenging, antioxidant, antimicrobial, anti-inflammation, antiplasmodial, antityrosinase, platelet aggregation, wound healing, anti-obesity, disease and insect resistant activities as well as enhancing percutaneous penetration.

### General Botanical Description

According to the description of Flora Republicae Popularis Sinicae and Chinese Materia Medica [19,20], *B. balsamifera* is a perennial herb or subshrub, which rises about 1–3 meters in height. Its stem is strong and taupe, and erects with taupe, longitudinal edges. Its upper internodes are covered by dense tawny nonglandular hair. Its leaves, when triturated, send out a unique, cool aroma, which can make people feel refreshed. The leaves are wide ovoid or oblong-lanceolate in shape at the bottom, 22–25 cm in length, 8–10 cm in width. Its base is attenuated with petiole, narrow linear appendants of 3–5 pairs on both sides, pubescenced above, slight brown or thick yellow-white silky-villous, highlighted below midrib, with lateral veins of 10–15 pairs. The leaves at the top are oblong-lanceolate or ovate-lanceolate in shape, 7–12 cm in length, 1.5–3.5 cm in width, with an acuminate apex, a slightly acuminate base, without petiole or with a short petiole with narrow linear appendants of 1–3 pairs, entire or with thin serration or pinnatopectinate. Capitulum is arranged in much more branched leafy panicles; a peduncle with yellow and dense pubescence; an involucre campanulate, and a dense pubescence at the back. Its flower is yellow, with numerous female parts; tubular corolla, receptacle honeycomb, and corolla thin tubulous. Akene is cylindrical, with five edges, a dense pubescence, and a red-brown rough hairy pappus. The flowering period almost covers the whole year. *B. balsamifera* often grows in forest edges, under forests, river beds, valleys, or grasslands, and the altitude is 600–1000 m. In addition to its various Chinese locations it is also distributed in India, Pakistan, Burma, Indo-China Peninsula, Malaysia, Indonesia and Philippines.

## 2. Phytochemistry

There have been more than 100 volatile or non-volatile constituents isolated from sambong, including monoterpenes, sesquiterpenes, diterpenes, flavonoids, organic acids, esters, alcohols, dihydroflavone, and sterols. The study of the plant mainly focused on the volatile oils and flavonoids, which possessed various bioactivities *in vivo* and *in vitro*. The chemical constituents of *B. balsamifera* have been reviewed earlier [8,18].

### 2.1. Volatile Constituents

The volatile constituents account for the largest amount of the constituents in *B. balsamifera*, which are the major active constituents containing terpenoids, fatty acids, phenols, alcohols, aldehydes, ethers, ketones, pyridines, furans, and alkanes (Table 1, Appendix A). The most important constituent is l-borneol. To address the need for a natural borneol, different extraction methods have been used to extract the volatile constituents from *B. balsamifera*. Steam distillation (SD), simultaneous distillation and extraction (SDE), and CO_2_ supercritical extraction were the most common methods in the extraction of volatile oils [21,22,23,24]. Wang *et al.* have adopted SD, SDE, and headspace solid-phase micro-extraction (HS-SPME) in order to obtain the volatile compounds of *B. balsamifera* leaves [23]. The extracts were then isolated and were identified by gas chromatography mass spectrometry (GC-MS). They found that alcohols and terpenoids were the main volatile compounds and the terpenoids accounted for a considerable proportion. Fifty, twenty-four, and forty-nine kinds of compounds were extracted by SD, SDE, and HS-SPME, respectively.

**Table 1:** The known volatile constituents of *B. balsamifera*.

Classification		Compound name	Molecular formula	References
Monoterpenes	1	l-borneol	C_10_H_18_O	[22,25,26]
	2	Isoborneol	C_10_H_18_O	[22,23,27]
	3	(+)-Limonene	C_10_H_16_	[21,25,28]
	4	(−)-Limonene	C_10_H_16_	[23]
	5	(E) Ocimene	C_10_H_16_	[23,27,28]
	6	(Z)-*β*-Ocimene	C_10_H_16_	[23]
	7	*β*-Myrcene	C_10_H_16_	[28]
	8	Camphene	C_10_H_16_	[21,23,25,27,28]
	9	*α*-Pinene	C_10_H_16_	[21,23,25,27,28]
	10	*β*-Pinene	C_10_H_16_	[21,23,25,27,28]
	11	Terpinen-4-ol	C_10_H_18_O	[21,22,27]
	12	Perillyl alcohol	C_10_H_16_O	[21,28]
	13	Chrysanthenone	C_10_H1_4_O	[21,23,27]
	14	*α*-Terpineol	C_10_H_18_O	[22,23,26,27]
	15	Bornyl acetate	C_12_H_20_O_2_	[23]
	16	Sabinene	C_10_H_16_	[27]
	17	*α*-Thujene	C_10_H_16_	[27]
	18	Trans-linalool oxide	C_10_H_18_O_2_	[27]
	19	Linalooloxide	C_10_H_18_O_2_	[21]
	20	Camphor	C_10_H_16_O	[21,22,25,26,27,28]
	21	1,8-Cineole	C_10_H_18_O	[26]
Oxygenated monoterpenes	22	Perilla aldehyde	C_10_H_14_O	[21,25,28]
	23	Cuminaldehyde	C_10_H_12_O	[27,28]
	24	Myrtenal	C_10_H_14_O	[21,27]
	25	Thymohydroquinone dimethyl ether	C_12_H_18_O_2_	[21]
Sesquiterpenes	26	*α*-Gurjunene	C_15_H_24_	[21,23,25,27]
	27	Alloaromadendrene	C_15_H_24_	[23,25]
	28	(+)-Aromadendrene	C_15_H_24_	[23]
	29	Aromadendrene	C_15_H_24_	[21,23,27,28]
	30	Aromadendrene oxide	C_15_H_24_O	[28]
	31	Aromadendrene, dehydro	C_15_H_22_	[28]
	32	Longifolene	C_15_H_24_	[23,27]
	33	*α*-Caryophyllene	C_15_H_24_	[21,23,25,26,27,28]
	34	*β*-Caryophyllene	C_15_H_24_	[21,25,27]
	35	Caryophyllene oxide	C_15_H_24_O	[21,22,26,27,28]
	36	Guaia-3,9-diene	C_15_H_24_	[26,28]
	37	*γ*-Cadinene	C_15_H_24_	[21,27]
	38	*δ*-Cadinene	C_15_H_24_	[21,27,28]
	39	*β*-Selinene	C_15_H_24_	[27]
	40	(+)-*β*-Selinene(*β*-Selinene)	C_15_H_24_	[26]
	41	*β*-Gurjunene	C_15_H_24_	[26]
	42	(+)-*γ*-Gurjunene	C_15_H_24_	[23]
	43	Thujopsene-13	C_15_H_24_	[23,28]
	44	*β*-Elemene	C_15_H_24_	[28]
	45	(−)-*β*-Elemene	C_15_H_24_	[23]
	46	(−)-*γ*-Cadinene	C_15_H_24_	[23]
	47	(−)-*δ*-Cadinene	C_15_H_24_	[23]
	48	10-Epi- *γ*-Eudesmol	C_15_H_26_O	[21,23,25,27]
	49	Globulol	C_15_H_26_O	[27,28]
	50	(−)-Guaiol	C_15_H_26_O	[21,22,25,28]
	51	Ledol	C_15_H_26_O	[21,23,25,28]
	52	*γ*-Muurolene	C_15_H_24_	[26,28]
	53	Elemol	C_15_H_26_O	[21,23,27]
	54	*α*-Eudesmol	C_15_H_26_O	[21,25]
	55	*β*-Eudesmol	C_15_H_26_O	[21,23,27,28]
Sesquiterpenes	56	*γ*-Eudesmol	C_15_H_26_O	[21,23,25,28]
	57	Carotol	C_15_H_26_O	[23,28]
	58	Cubenol	C_15_H_26_O	[23,28]
Diterpenes	59	16-Kaurene	C_20_H_32_	[23]
	60	1-Ang-4,7-dihydroxyeudesmane	C_20_H_34_O_4_	[9]
	61	Phytol	C_20_H_40_O	[26,28]
	62	Blumeaene A	C_20_H_30_O_5_	[9]
	63	Blumeaene B	C_20_H_30_O_5_	[9]
	64	Blumeaene C	C_19_H_30_O_5_	[9]
	65	Blumeaene D	C_21_H_32_O_5_	[9]
	66	Blumeaene E	C_20_H_30_O_6_	[9]
	67	Blumeaene F	C_20_H_30_O_6_	[9]
	68	Blumeaene G	C_20_H_30_O_5_	[9]
	69	Blumeaene H	C_19_H_30_O_5_	[9]
	70	Blumeaene I	C_20_H_30_O_6_	[9]
	71	Blumeaene J	C_20_H_32_O_7_	[9]
Fatty acids	72	(11Z)-11-hexadecenoic acid	C_16_H_30_O_2_	[23]
	73	Trans-2-undecenoic acid	C_11_H_20_O_2_	[22]
	74	9-Hexadecenoic acid	C_16_H_30_O_2_	[27]
	75	Capric acid	C_10_H_20_O_2_	[27]
	76	Palmitic acid	C_16_H_32_O_2_	[23]
Phenols	77	Xanthoxylin	C_10_H_12_O_4_	[23,29,30]
	78	Eugenol	C_10_H_12_O_2_	[21,22,26]
	79	Dimethoxydurene	C_12_H_18_O_2_	[25]
Alcohols	80	1-Octen-3-ol	C_8_H_16_O	[21,23,25,27]
	81	3-Octanol	C_8_H_18_O	[21,23,25]
Aldehydes	82	3-Propyl benzaldehyde	C_10_H_12_O	[25,31]
	83	4-Isopropylbenzaldehyde	C_10_H_12_O	[21,22]
Ketones	84	3-Octanone	C_8_H_16_O	[21,23]
	85	2-Hydroxy-4,6-dimethoxyacetophenone	C_10_H_12_O_4_	[27]
Pyridines	86	3-Fluoro-5-amino-pyridine	C_5_H_5_FN_2_	[22]
Furans	87	Furan,4,5-diethy-2,3-dihydro-2,3-dimethyl	C_10_H_18_O	[22]
	88	1,3,4,5,6,7-hexahydro-2,5,5-trimete-hyl-2H-2,4A-et hanonaphthalene	C_10_H_14_	[23,25]
Others	89	Paracymene (4-Isopropyltoluene)	C_10_H_14_	[27]
	90	3-Nitrophthalic acid	C_8_H_3_NO_6_	[23]
	91	*α*-Butenoic acid, 3-methoxy-4-nitro	C_5_H_7_NO_5_	[22]
	92	*O*-acetyl-1-serine	C_5_H_9_NO_4_	[22]
	93	4,4-dimethyltetracyclo[6.3.2.0^2,5^.0^1.8^]tridecan-9-ol	C_13_H_20_O	[28]

Several volatile oils are contained in the leaves and branchlets of *B. balsamifera*, which are the key crude materials of refined borneol [28]. Volatile oil in *B. balsamifera* is a yellow oily liquid with a unique aroma [32]. The oil yield of this plant is at least 2.5 mg/g (DW) in Guizhou, China. If the plants are fertilized in certain way, it can be much higher, may be up to 7.72 mg/g (DW) [33]. In Hainan (China), Pang *et al.* also got an oil yield of 3.2–4.3 mg/g (unpublished data). Some previous studies have reported that the volatile oils of *B. balsamifera* mainly contained monoterpenes and sesquiterpenes, such as l-borneol, 10-epi-γ-eudesmol, γ-eudesmol, β-eudesmol, α-eudesmol, limonene, l-camphor, palmitic acid, and d-camphor [21]. Hao *et al.* have qualitatively and quantitatively analyzed the volatile oil of *B. balsamifera* growing in Guizhou Province by GC-MS [25]. A total of 28 constituents were identified in this study, including: l-borneol, β-caryophyllene, camphor, γ-eudesmol, 1-octen-3-ol, trans-β-ocimene, and 1,3,4,5,6,7-hexahydro-2,5,5-trimethyl-2*H*-2,4a-ethanonaphthalene. Fifty-six compounds were separated and identified in the volatile oils of *B. balsamifera* by GC-MS according to the studies of Du *et al.* [22]. According to their studies, the main constituents are borneol and camphor. Others are isoborneol, terpineol, caryophyllene, eugenol, guaiol, and cubenol. Bhuiyan *et al.* also studied the volatile oils of *B. balsamifera* leaves to isolate fifty constituents, which contributed to 99.07% of the oil [28]. The main constituents were borneol (33.22%), caryophyllene (8.24%), ledol (7.12%), and 4,4-dimethyltetracyclo[6.3.2.0^2,5^.0^1,8^]tridecan-9-ol, (5.18%). It was difficult to give a trivial name to or classify 4,4-dimethyltetracyclo-[6.3.2.0^2,5^.0^1,8^]tridecan-9-ol, because it had a non-typical structure, which made it seem rather a fragment of some other compound(s). The contents of other constituents such as phytol, caryophyllene oxide, thujopsene-13, guaiol, dimethoxydurene, and γ-eudesmol were 3%–5%. Wen *et al.* adopted a RP-HPLC method to measure the content of xanthoxylin in different parts of *B. balsamifera* [34]. Xia *et al.* selected fourteen different regions of *B. balsamifera* to establish a GC chromatographic fingerprint [35]. The sample plants were selected from Hongshuihe village (II), Luodian, and Guizhou with the highest concentration of l-borneol. Chu *et al*. reported 1,8-Cineole contribute 20.98% to *B. balsamifera* oil in Nanning, Guangxi Zhuang Autonomous Region, China, while Pang *et al*., didn’t find it when they analyzed sambong oil with GC-MS (unpublished data), as well as some other research [22,25]. Sun, Shirota, and Xu also determined the contents of constituents in *B. balsamifera* oil [36,37,38]. The Aifen, *B. balsamifera* powder, is a crude product during the production of Aipian. Jiang *et al.* found that the yield and extraction rate of Aifen increased significantly if the plant materials were harvested from October to December [39].

### 2.2. Non-Volatile Constituents

#### 2.2.1. Flavonoids Constituents

Flavonoids, including flavonoid, flavanone and chalcone constituents, are the major non-volatile constituents of *B. balsamifera* (Table 2, Appendix B). The ultrasonic extraction methods of total flavonoids of *B. balsamifera* were studied by Pang *et al.* [40]. It was found that 30% ethanol was the suitable extraction solvent with the solid/liquid ratio of 1/300. It was then extracted by ultrasonic frequency (85 Hz), twice, each time for 30 min, where the extraction yield was 208.6 mg/g. Previous phytochemical studies have shown that the leaves of *B. balsamifera* included a number of flavonoids, such as blumeatin, velutin, tamarixetin, dihydroquercetin-7,4'-dimethyl ether, ombuine, rhamnetin, luteolin-7-methyl ether, luteolin, quercetin, 5,7,3',5'-tetrahydroxyflavanone, and dihydroquercetin-4'-methyl ether [16,41,42,43,44]. With the advances in phytochemical studies of *B. balsamifera*, flavonoids have been recognized for their medicinal properties and the investigation, validation, standardization of the local plant have been developed as an herbal medicine. Ali *et al.* extracted 3,4',5-trihydroxy-3',7-dimethoxyflavanone from the ligroin extract of *B. balsamifera* leaves [31]. The constituents of 3',4',5-trihydroxy-7-methoxyflavanone and a new bioflavonoid, determined as 3-O-7''-biluteolin, were extracted by acetone. Zhu *et al.* isolated three constituents, including: xanthoxylin, blumeatin, dihydroquercetin-7, and 4'-dimethylethe [29]. Chen *et al.* also obtained three flavonoid compounds from aerial parts of *B. balsamifera* [45]. The compounds of 5,7-dihydroxy-3,3',4'-trim ethoxy flavone, davidigenin, catechin, ayanin, and davidioside were originally extracted from *B. balsamifera*. In the same year, Huang *et al.* isolated and identified seven constituents from this plant [46]. The constituents of 5,4'-dihydroxy-7-methoxyflavone and 5,4'-dihydroxy-3,3',7-trimethoxy flavanone were first found in the plant. Deng *et al.*, Zhu, Saewan *et al.*, Yan *et al.*, and Tan *et al.* also studied the flavonoid constituents [14,29,47,48,49]. Liang *et al.* used the methods of silica gel column chromatography, TLC, and Sephadex LH-20 column chromatography in order to isolate the chemical constituents of the plant, the structures of which were determined by physico-chemical constants and spectral analysis [39]. As a result, seven constituents were isolated. Besides, the contents of the flavonoids were determined by different methods. The chromatographic methods of RP-HPLC (reversed-phase high-performance liquid chromatographic), and HPLC were the most widely used to determine the contents of flavonoids of *B. balsamifera* [44,50]. Huang *et al.* determined the total amount of flavonoids in different parts of *B. balsamifera* [51]. The results exhibited that the total flavonoid content was 2.94%, 1.21%, and 1.36% in the leaves, branch, and stem, respectively.

**Table 2:** The known non-volatile constituents of *B. balsamifera*.

Classification		Compound Name	Molecular Formula	References
Flavones	1	4',5-Dihydroxy-7-methyletherflavanone	C_16_H_12_O_5_	[30,46]
	2	Luteolin	C_15_H_10_O_6_	[9,14,30,39,46]
	3	Luteolin-7-methyl-ether	C_16_H_12_O_6_	[14,30,46]
	4	Diosmetin (Luteolin 4'-methyl ether)	C_16_H_12_O_6_	[48]
	5	Chrysoeriol (Luteolin 3'-methyl ether)	C_16_H_12_O_6_	[48]
Flavonols	6	Quercetin	C_15_H_10_O_7_	[9,14,39,44]
	7	3,5,3',4'-Tetrahydroxy-7-methoxyflavone	C_16_H_12_O_7_	[9,31,47]
	8	3,5,3'-Trihydroxy-7,4-dimethoxyflavone	C_17_H_14_O_7_	[9,45,47]
	9	Rhamnetin (7-Methoxyquercetin)	C_16_H_12_O_7_	[14,30,46]
	10	Tamarixetin	C_16_H_12_O_7_	[14]
	11	Ombuine	C_17_H_14_O_7_	[49]
	12	3,5,7-Trihydroxy-3'4'-dimethoxyflavone	C_17_H_14_O_7_	[48]
	13	3,3',4',5-Tetrahydroxy-7-methoxyflavone	C_16_H_15_O_6_	[48]
	14	3,5-Dihydroxy-3',4',7-trimethoxyflavone	C_18_H_16_O_7_	[48]
	15	4',5-Dihydroxy-3,3',7-trimethoxy flavanone	C_18_H_16_O_7_	[30,46]
	16	5,7-Dihydroxy-3,3',4',-trimethoxyflavone	C_18_H_16_O_7_	[9,45]
	17	Ayanin	C_18_H_16_O_7_	[9,45]
	18	Chrysosplenol C	C_18_H_16_O_8_	[45]
	19	4',5,7-Trihydroxy-3,3'-dimethoxyflavone	C_17_H_14_O_7_	[48]
	20	Hyperoside	C_21_H_20_O_12_	[48]
	21	Isoquercitrin	C_21_H_20_O_12_	[48]
Flavanones	22	Blumeatin (5,3',5'-trihydroxy-methoxydihydro-flavone)	C_16_H_14_O_6_	[9,14,29,30,45,52]
	23	Eriodictyol	C_15_H_12_O_6_	[30,46]
	24	5,7,3',5'-Tetrahydroxyflavanone	C_15_H_12_O_6_	[9,44,45]
	25	3',4',5-Trihydroxy-7-metoxyflavanone	C_16_H_15_O_8_	[45]
Flavanonols	26	Dihydroquercetin-4'-methylether	C_16_H_14_O_7_	[9,12,14,30,39,45,52,53]
	27	Dihydroquercetin-7,4'-dimethylether	C_17_H_16_O_7_	[9,14,29,30,39,45,52,53]
	28	3,4'5-Trihydroxy-3'7-dimethoxyflavanone	C_17_H_18_O_9_	[45]
	29	3,3',5,5',7-Pentahydroxyflavanone	C_15_H_12_O_7_	[48]
	30	3,3',4',5-Tetrahydroxy-7-methoxyflavanone	C_16_H_15_O_8_	[48]
	31	3,3',5-Trihydroxy-4',7-dimethoxyflavanone	C_17_H_18_O_9_	[48]
	32	3,3',5,7-Tetrahydroxy-4'-methoxyflavanone	C_16_H_15_O_8_	[48]
	33	3',4',5-Trihydroxy-3,7-dimetoxyflavanone	C_17_H_18_O_9_	[48]
Flavanols	34	Catechin	C_15_H_14_O_6_	[9,45]
	35	(2R,3R)-(+)-7-*O*-Methyldihydroquercetin	C_16_H_14_O_7_	[53]
Chalcones	36	Davidioside	C_21_H_24_O_9_	[9,45]
	37	Davidigenin	C_15_H_14_O_4_	[9,45]
Sesquiterpene lactone	38	Blumealactone A	C_19_H_26_O_6_	[54]
	39	Blumealactone B	C_20_H_28_O_6_	[54]
	40	Blumealactone C	C_16_H_22_O_6_	[54]
Sterides	41	*β*-Sitosterol	C_29_H_50_O	[9]
	42	5 *α*,8*α*-Epidioxyergosta-6,22-dien-3*β*-ol	C_28_H_44_O_3_	[9]
	43	Daucosterol	C_35_H_60_O_6_	[9,39]
Diterpenes	44	Cryptomeridiol	C_15_H_28_O_2_	[55]
Triterpenes	45	3,13-Clerodadiene-6,15-diol	C_20_H_34_O_2_	[9]
	46	Austroinulin	C_20_H_34_O_3_	[55]
Lignans	47	Syringaresinol	C_22_H_26_O_8_	[9]
Coumarin	48	Hydranngetin	C_10_H_8_O_4_	[9]
	49	Umberlliferone (7-hydroxycoumarin)	C_9_H_6_O_3_	[9]
Naphthalenone	50	5,7-Dihydroxychromone	C_9_H_6_O_4_	[49]

#### 2.2.2. Sterols

Apart from the above constituents, a small number of sterols were also isolated from *B. balsamifera*. Zhao *et al.* obtained colorless acicular as well as sheet crystals from *B. balsamifera* by silica gel column chromatography [56], where the crystals were identified to be stigmasterol and β-sitosterol by TLC and melting point measurement. Chen isolated β-sitosterol, daucosterol, and 5α,8α-epidioxyergosta-6,22-dien-3β-ol from the aerial sections of *B. balsamifera* collected from Mengla, Yunnan by MS determination [9]. Liang *et al.* yielded seven compounds, which were isolated from ethyl acetate and chloroform extract, including daucosterol [39].

#### 2.2.3. Sesquiterpene Lactone (SLs)

Sesquiterpene lactones (SLs) are a group of common chemicals in many Asteraceae plants. They were famous because they had cytotoxic and potential to be tumor inhibitors [57,58]. In sambong, a member of Asteraceae family, there were three sesquiterpene lactones, Blumealactone A, Blumealactone B, and Blumealactone C. Fujimoto *et al.* isolated them by extracting its dried leaves with 90% ethanol [54].

#### 2.2.4. Other Constituents

There were some other constituents in this plant. Chen found two coumarin constituents, such as umberlliferone and hydranngetin, in *B. balsamifera*. He also found a lignans constituent, which was syringaresinol [9].

## 3. Biological Activities

### 3.1. Antitumor Activity

Hasegawa *et al.* extracted a dihydroflavonol from *B. balsamifera* as a result of screening among more than 150 plant materials [12]. The dihydroflavonol components showed the most significant synergism with tumor related apoptosis inducing ligand (TRAIL). It enhanced the level of TRAIL-R2 promoter activity and promoted the expression of surface protein in a p53-independent manner. The ethanol extract of *B. balsamifera* leaves was tested on male mice to investigate its hepatoxicity. The results exhibited that the hepatic cells, sitplasm, nucleus, and sinusoid of the mice liver were damaged through some changes in the liver color and texture [59]. The methanol extract of *B. balsamifera* inhibited the growth in rat and showed no cytotoxicity on human hepatocellular carcinoma cells. The methanol extract decreased the expression of cyclin-E and phosphorylation of retinoblastoma (Rb) protein resulting in cell cycle arrest. Likewise, it decreased the level of the proliferation related ligand (APRIL) [60,61]. Moreover, the methanol extract of *B. balsamifera* was used to determine its cytotoxicity on a panel of human cancer cell lines by MTT assay. There was no regular or acute cytotoxicity on the cells of HepG2, HCT-116, T-47D, NCl-H23 and CCD-18Co [62]. Saewan *et al.* found six compounds out of nine isolated flavonoids to have cytotoxicity against KB, MCF-7, and NCI-H187 cancer cell lines [14]. These six compounds were evaluated for cytotoxicity against KB, MCF-7, and NCI-H187 cancer cell lines. Three compounds were active against the KB cells with the IC_50_ values of 17.09, 47.72, and 17.83 µg/mL, respectively. Another three compounds exhibited a moderate activity against the NCI-H187 cells with the IC_50_ values of 16.29, 29.97, and 20.59 µg/mL. Luteolin-7-methyl ether showed a strong cytotoxicity against human lung cancer (NCI-H187) cell lines with an IC_50_ of 1.29 µg/mL and a moderate toxicity against oral cavity cancer (KB) cell lines with an IC_50_ of 17.83 µg/mL. Li *et al.* studied the antitumor activity determined by means of 3-(4,5-dimethylthiazol-2-yl)-2,5-diphenyltetrazolium bromide assay [13]. The three endophytic streptomycetes strains of *B. balsamifera*, including: YIM 56092, YIM 56093, and YIM 56099 exhibited anticancer activity. Yet, different strains displayed different antitumor activities. The YIM 56092 strain displayed a cytotoxic activity on polyketide synthases I (PKS-I) nonribosomal peptide synthetases (NRPS) and P388D1. The YIM 56093 strain displayed a cytotoxic activity on PKS-Ⅱ, NRPS, and P388D1. The YIM 56099 was on the PKS-I, PKS-II, and NRPS. Fuijimoto *et al.*, extracted blumealactone A, B, and C from sambong’s dried leaves and found them could inhibit the growth of Yoshida sarcoma at the concentration of 5–10 μg/ml [54]. Lee disclosed a medication combination including sambong (Ainaxiang) and found it could enhance the efficiency of curing hepatoma and pancreatic cancer treatments [63].

### 3.2. Hepatoprotective Effects

Xu *et al.* demonstrated that oral blumeatin (5,3',5-trihydroxy-7-methoxydihydroflavone) exhibited a significant protective activity against the liver injury caused by paracetamol and prednisolone [64]. Furthermore, Xu and Zhao have shown that five other *blumea* flavanones possessed protective activity for acute experimental liver injury [65]. Pu *et al.* further verified the five *blumea* flavanones protecting the hepatocytes against lipid peroxidation, which was induced by CCl_4_ or FeSO_4_+cysteine. Certain concentration of the five compounds (10–100 μmol/L) inhibited the malonaldehyde production, GSH depletion, and GPT leakage of hepatocytes [66]. Furthermore, *blumea* flavanone II showed the strongest activity. They also reported that the *blumea* flavones had protective effects against acute liver injury induced by different chemicals [67].

### 3.3. Superoxide Radical Scavenging Activity

The methanol extracts of *B. balsamifera* leaves showed a higher radical scavenging activity than the chloroform extracts. However, the pet-ether extracts had less activity against nonenzymatically generated superoxide radicals. The capacity of nine kinds of flavonoids (100 mmol/L) of the plant was decreased as follows: quercetin > luteolin > 5,7,3',5'-tetrahydroxyflavanone > blumeatin > rhamnetin > tamarixetin > luteolin-7-methyl ether > dihydroquercetin-4'-methyl ether > dihydroquercetin-4',7-dimethyl ether. The flavonoids showed more activity than methylated compounds [16]. Zhao and Xu studied antiperoxidant activities of five compounds of *blumea* flavanones [68]. As a result, five compounds with the concentration of 10^−5^–10^−4^ mol/L of blocked malondialdehyde formation in homogenates and in liver mitochondria of rats *in vitro*.

### 3.4. Antioxidant Activity

Nguyen *et al.* demonstrated the methanol extracts of *B. balsamifera* (collected in Lam Dong province) with strong xanthine oxidase inhibitory activity with an IC_50_ value of 6.0 μg/mL [69]. They also verified the seven compounds of *B. balsamifera* methanol extract in Vietnam, which exhibited significant xanthine oxidase inhibitory activity. Three compounds among them, such as (2*R*,3*S*)-(−)-4'-*O*-methyldihydroquercetin, quercetin, and quercetin-3,3',4', showed a higher potent inhibitory activity, with their IC_50_ values ranging from 0.23 to 1.91 mmol/L, as compared to that of the positive control allopurinol (IC_50_ of 2.50 mmol/L) [70]. Furthermore, Nessa *et al.* have found that the methanol extract of *B. balsamifera* exhibited a higher xanthine oxidase inhibitory activity as compared to that of the chloroform and pet-ether extracts [71]. The activity of the isolated flavonoids’ order was as follow: allopurinol > luteolin > quercetin > tamarixetin > 5,7,3',5'-tetrahydroxyflavanone > rhamnetin > luteolin-7-methylether > blumeatin > dihydroquercetin-4'-methylether > dihydroquercetin-7,4'-dimethyl ether > l-ascorbic acid. The plant of *B. balsamifera* was collected in Taiwan and the dry powder of the whole plant was extracted twice with methanol. The methanol extracts showed an activity on scavenging 1,1-diphenyl-2-picrylhydrazyl (DPPH) radicals with an IC_50_ value of 72 g/mL. The superoxide anion scavenging activity was over 200 g/mL [72].

### 3.5. Anti-Microbial and Anti-Inflammation Activity

Ongsakul *et al.* claimed that the crude aqueous and ethanolic extracts of *B. balsamifera* displayed no significant antibacterial activity against the strains of *Staphylococcus aureus* and *Escherichia coli* [73]. However, the stain of *B. balsamifera*, including YIM 56092 and YIM 56093, displayed a significant activity against *S. epidermidis*, such that YIM 56099 was active against *E. coli*. There seems to be no antimicrobial activity against *S. aureus*, *Klebsiella pneumonia*, and *Candida albicans* [13]. Chen isolated twelve new compounds [9], four of which displayed inhibitory activities against LPS-induced NO production in RAW 264.7 with the IC_50_ values of 40.06, 46.35, 57.80, and 59.44 μg/mL, respectively. Sakee *et al.* reported the essential oil of *B. balsamifera* to have a minimum inhibitory concentration (MIC) of 150 µg/mL and 1.2 mg/mL against *Bacillus cereus*, *S. aureus* and *Candida albicans*, respectively [74]. Furthermore, the hexane extract inhibited *Enterobacter* *cloacae* and *S. aureus*. These results suggested that the extracts of *B. balsamifera* possessed an activity against certain kinds of infectious and toxin-producing microorganisms. It could potentially be utilized to prevent and treat microbial diseases.

### 3.6. Antiplasmodial Activities

According to the traditional efficacy of relieving fever, the methanol extract of *B. balsamifera* from Forest Research Institute Malaysia was investigated for any potential antiplasmodial activity. The extracts of roots and stems exhibited some activity against *Plasmodium falciparum* D10 strain (sensitive strain) with an IC_50_ value of (26.25 ± 2.47) μg/mL and (7.75 ± 0.35) μg/mL, respectively [75].

### 3.7. Antityrosinase Activities

The ethylacetate extract consisting of nine flavonoids were isolated from the leaves of *B. balsamifera*. Their antityrosinase activities were surveyed by Saewan *et al.* [14]. According to their reports, compared with arbutin, two dihydroflavonols, dihydroquercetin-4'-methylether and dihydroquercetin-7,4'-dimethylether, and three flavonols, quercetin, rhamnetin and tamarixetin, showed a significantly higher inhibitory activity, but another two flavanones, 5,7,3',5'-tetrahydroxyflavanone and blumeatin, and two flavones, luteolin and luteolin-7-methyl ether, showed a lower inhibitory activity. The possible mechanism of the antityrosinase activity might be the cause of chelating with copper in the active center of tyrosinase.

### 3.8. Platelet Aggregation Activities

The concentration of 1.26 μmol/L blumeatin displayed a significant promoting activity on the rat and human platelet aggregation caused by arachidonic acid, 5-hydotypamice, and epinephrine. However, concentrations of 0.315 and 2.52 μmol/L inhibited platelet aggregation. It suggested that the effects of blumeatin on the platelet aggregation were dependent upon the concentration used. The injection of *B. balsamifera* extracts decreased the blood pressure, expanded the blood vessels, and inhibited the sympathetic nervous system in order to address the high pressure and insomnia. The infusion of the plant also had the function of diuresis [67].

### 3.9. Enhancing Percutaneous Penetration Activity

The l-borneol, as the main effective compound of *B. balsamifera*, showed a percutaneous penetration enhancer effect. The essential oil camphor and 1-menthol of the plant specifically promoted the percutaneous absorption of nicotinamide [9]. Fu *et al.* further verified the 0.5%, 1.0%, and 2.0% *B. balsamifera* oil enhancing albuterol sulfate transdermal absorption, respectively [76]. The percutaneous penetration of a combination of 1.0% *B. balsamifera* oil and 1.0% azone was less than that of their separate uses.

### 3.10. Wound Healing Activity

Wang *et al.* discovered that the external application of *B. balsamifera* oil on the intact and damaged skin of rats exhibited no acute toxicity [77]. The rats with pure *B. balsamifera* oil exposure at dosage of 2000 mg/kg for 24 h showed no allergic reaction or acute toxicity reaction, but a better wound recovery activity as compared to the one treated with non-*B. balsamifera* oil formulations. The results were consistent with the traditional use in ethnic minority, Li and Miao, in China in order to heal the skin wound and itch [19].

### 3.11. Anti-Obesity Activity

Kubota *et al.* reported that the extracts of *B. balsamifera* inhibited the lipid accumulation and glycerol-3-phosphate dehydrogenase (GPDH) activities [17], which mainly decreased the expressions of key adipogenic transcription factors, such as peroxisome proligerator-activated receptor γ, CCAAT element binding protein, and leptin in the 3T3-L1 adipocytes. Therefore, the extracts of *B. balsamifera* might possess antidiabetic, antiatherogenic, and anti-inflammatory functions. The methanol extracts of *B. balsamifera* (100 μg/mL) were used to investigate the ability of inhibiting blood vessel’s formation by the method of rat aortic ring. The results exhibited that there was no remarkable differences between *B. balsamifera* extracts and vehicle control treatment [62].

### 3.12. Disease and Insect Resistant Activity

Luo *et al.* reported that the acetone extracts of *B. balsamifera* possessed an activity against *Pryicutaria oryzae*, *Fusarium oxysporum* sp., *Colletorichum musae*, *C. gloeosporioides*, *C. capsici*, and *F. oxysporum* f. sp. *in vitro*, with an inhibition rate of over 90.0% [78]. The volatile oil of *B. balsamifera* inhibited *Aeromonas hydrophila*, *F. graminearum*, and *Magnaporthe grisea* [27]. Wang *et al.* also demonstrated that the extract of *B. balsamifera* leaves showed a 60.8% insecticidal activity against the adult *Aleurodicus disperses* [23]. Furthermore, the essential oil of *B. balsamifera* showed fumigant toxicity against the maize weevils, such as *Sitophilus zeamais* [26]. The crude oil also induced the death in the *S. zeamais* adults. The results suggested that the extracts of *B. balsamifera* possessed significant disease and insect resistant activities, and could be used as new potential plant pesticides.

## 4. Conclusions

In traditional medicine, the source of the plant should be primarily clear and definite. Thus, the general botanical description should be reviewed according to the *Flora Republicae Popularis Sinicae* and *Chinese Materia Medica* [19,20]. The herbal authentication of *B. balsamifera* was then further carried out to verify the plant source and medicinal efficacy [19]. These studies confirmed the accuracy and uniqueness of the source of *B. balsamifera*. In the same way, as a folk medicine, *B. balsamifera* has been widely used in South Asian countries, especially in China. Due to the chemical constituents contained in this plant for the different effects, the phytochemical research has been reviewed in this article. The chemical constituents were mostly volatile and non-volatile. The former constituents occupied the largest amount, which mainly included: terpenoids, fatty acids, phenols, alcohols, aldehydes, ethers, ketones, pyridines, furans, and alkanes. l-borneol was the most abundant and active constituent in this plant, while other constituents included flavonoids, sterols, sesquiterpene lactones, and other constituents [8,18]. The chemical composition of *B. balsamifera* oils varies from different plant populations [22,25,26,28], indicating more studies should be done on individual’s structural development, the cultivation, geographical, and climate conditions and essential oil standardization. The survey and summary of the extensive studies revealed that *B. balsamifera* was an essential and valuable medicinal plant used for folk treatments such as treating eczema, dermatitis, beriberi, lumbago, menorrhagia, rheumatism, skin injury, or used as insecticide [45]. As a traditional medicine, the biological and pharmacological studies of the plant materials, crude extracts, and isolated chemical constituents of *B. balsamifera* offered experimental and scientific proofs for its various traditional uses. The pharmacological studies focused on studying the anti-microbial and anti-inflammatory effects [13,15], antiplasmodial effects [75], platelet aggregation [79], wound healing [77], and disease and insect resistant activities [26], all of which confirmed the plant’s traditional uses. Moreover, some new pharmacological uses were discovered, such as antitumor [12,13,14], hepatoprotective [66], superoxide radical scavenging [16], antioxidant [69], antityrosinase [14], enhancing percutaneous penetration [76], and anti-obesity activities [17]. However, there was no experimental and pharmacological evidence to prove the traditional uses of this plant in rheumatism and lumbago. Besides, the isolated chemical constituent and its correspondent pharmacological effects were rarely simultaneously carried out in one study. Hasegawa *et al.* extracted a dihydroflavonol from *B. balsamifera*, which exhibited a significant synergism with TRAIL by pharmacological experiment [12]. Nevertheless, more studies should be done in abundance to understand the pharmacodynamic chemical constituents. The outcome from this study could establish the basis for its future clinical utilization in modern science.

On the basis of the above review, several prospects were revealed. In order to further define the effective chemical compounds, the biological activities of monomeric compounds, the plant material, and its crude extraction further studies were proposed. In addition, some traditional effects of *B. balsamifera*, such as rheumatism still need to be testified by more modern methods and further pharmacological trials. Few more aspects such as pharmacokinetics, molecular biology, and naturel medicinal chemistry should be utilized to study its phytochemical standardization and bioactivity identification according to its bioactive metabolism. Moreover, in the production process of TCM Aipian, *B. balsamifera* oil and Aifen could be important accessory substances yielded, which also possess many chemical and biological activities to be further studied in the future. Therefore, we concluded that the area of *B. balsamifera* research should be significantly expanded.

## Figures and Tables

**Appendix A:**
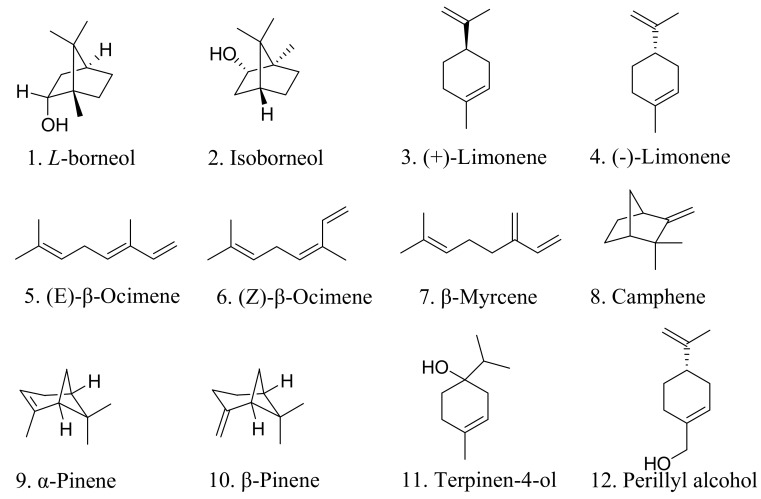

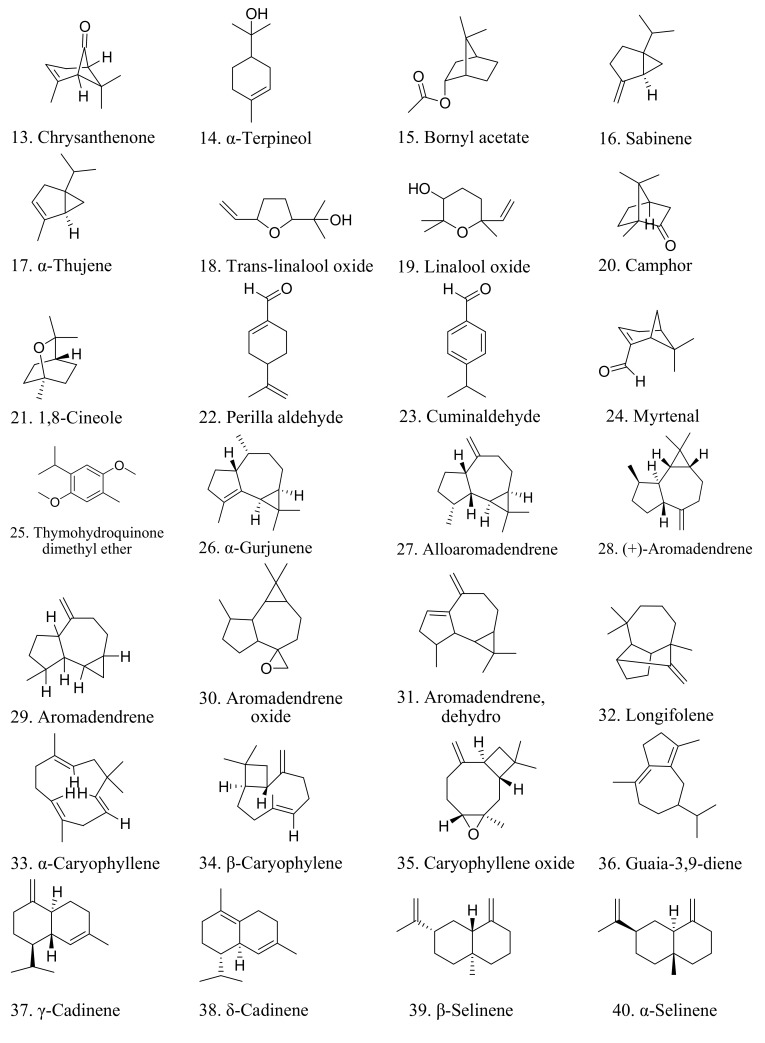

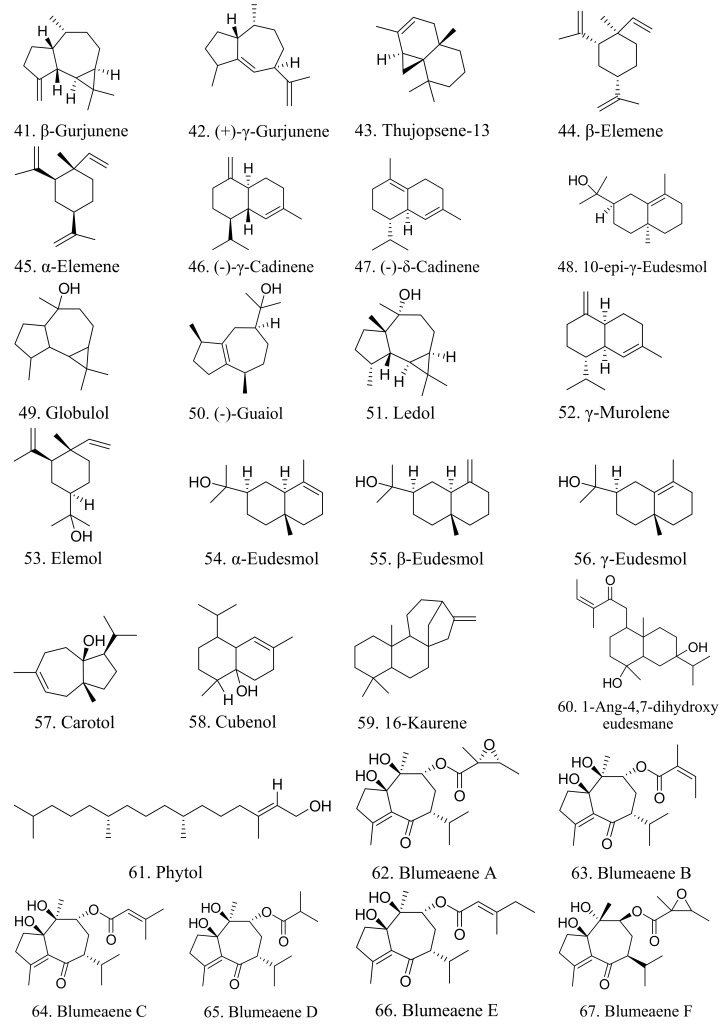

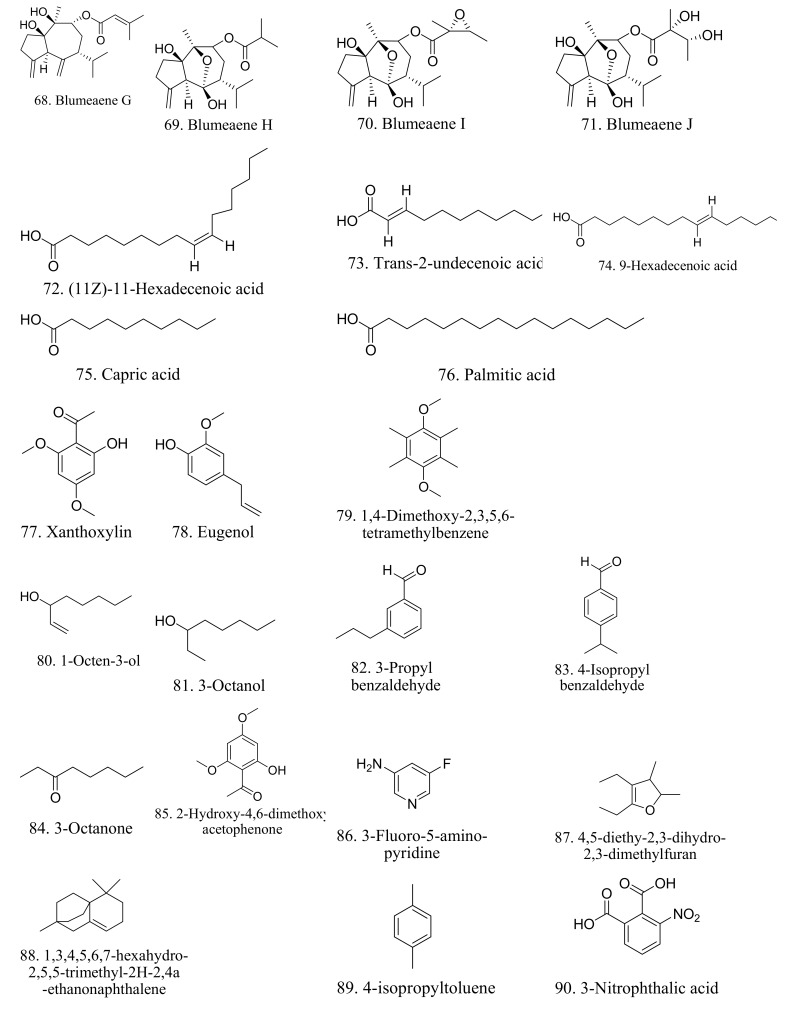

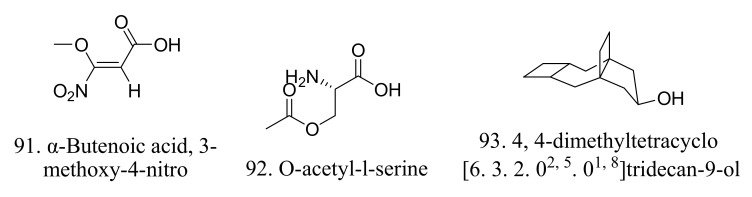
The structures of the volatile chemical constituents.

**Appendix B:**
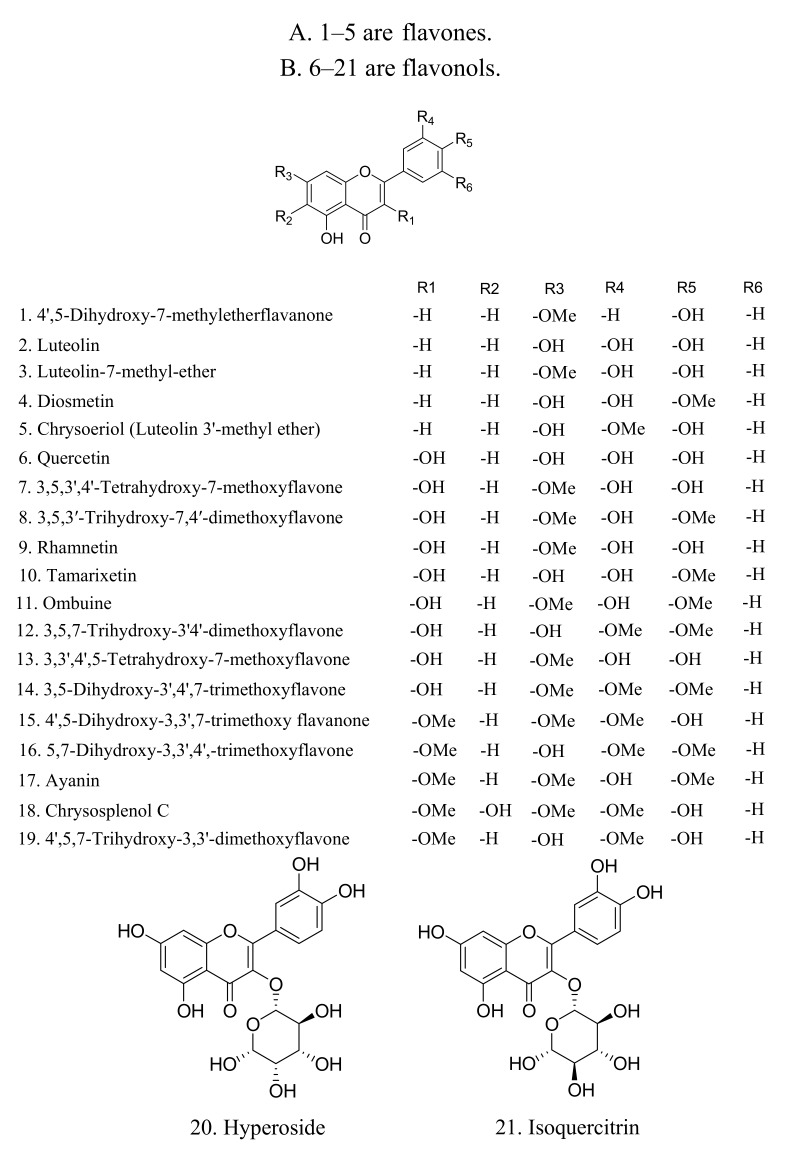

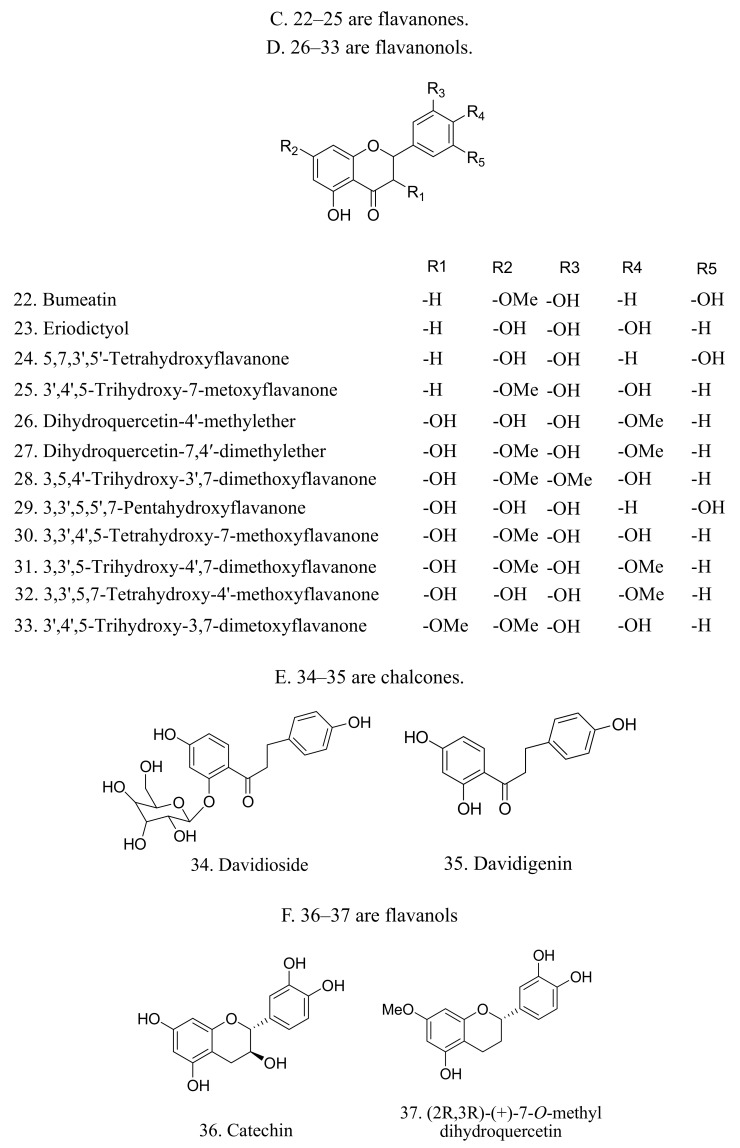

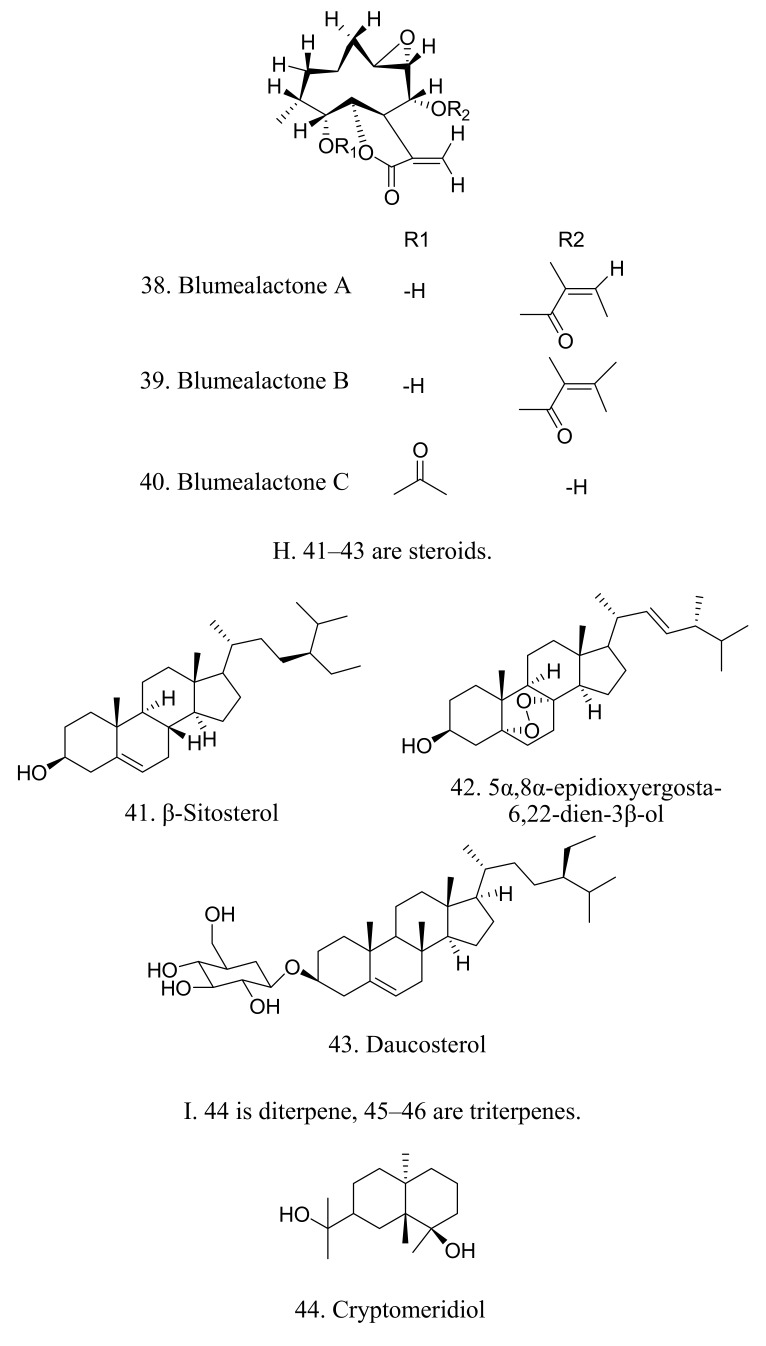

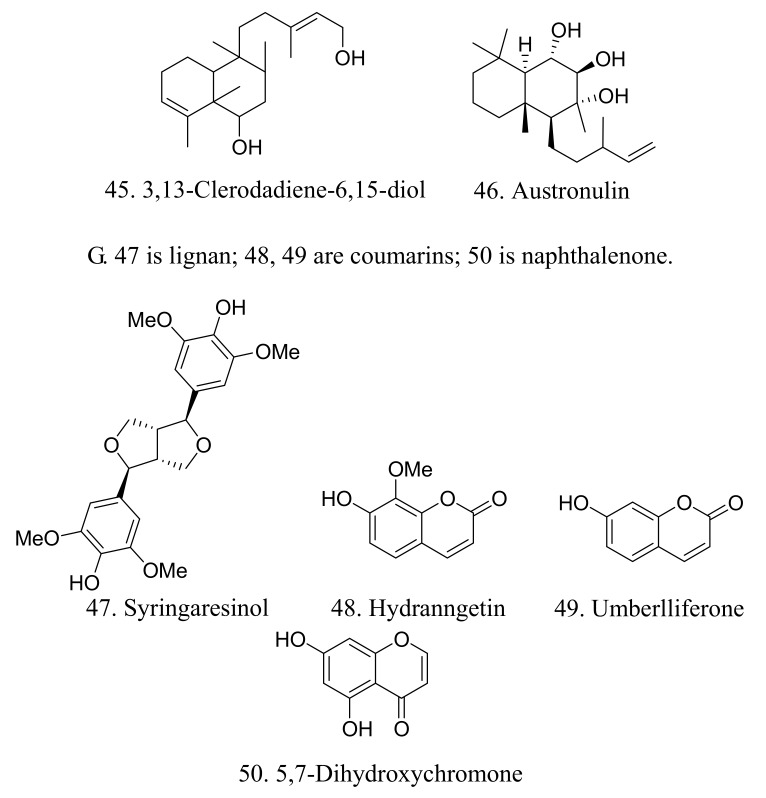
The structures of the non-volatile chemical constituents.
